# Supplementary material for: Saccharomyces boulardii (CNCM I-745) improves intestinal damage in sepsis by remodeling intestinal flora structure
Source: Front Cell Infect Microbiol. 2025 Jul 31;15:1602792. doi: 10.3389/fcimb.2025.1602792 (PMC12350329; doi:10.3389/fcimb.2025.1602792)
Supplement: Supplementary file 1 [file DataSheet1.zip › summary/summary/7_Differential_analysis/4_Manhattan_plot/Group.AW4_vs_AW0/Group.AW4_vs_AW0_manhattan.pdf]

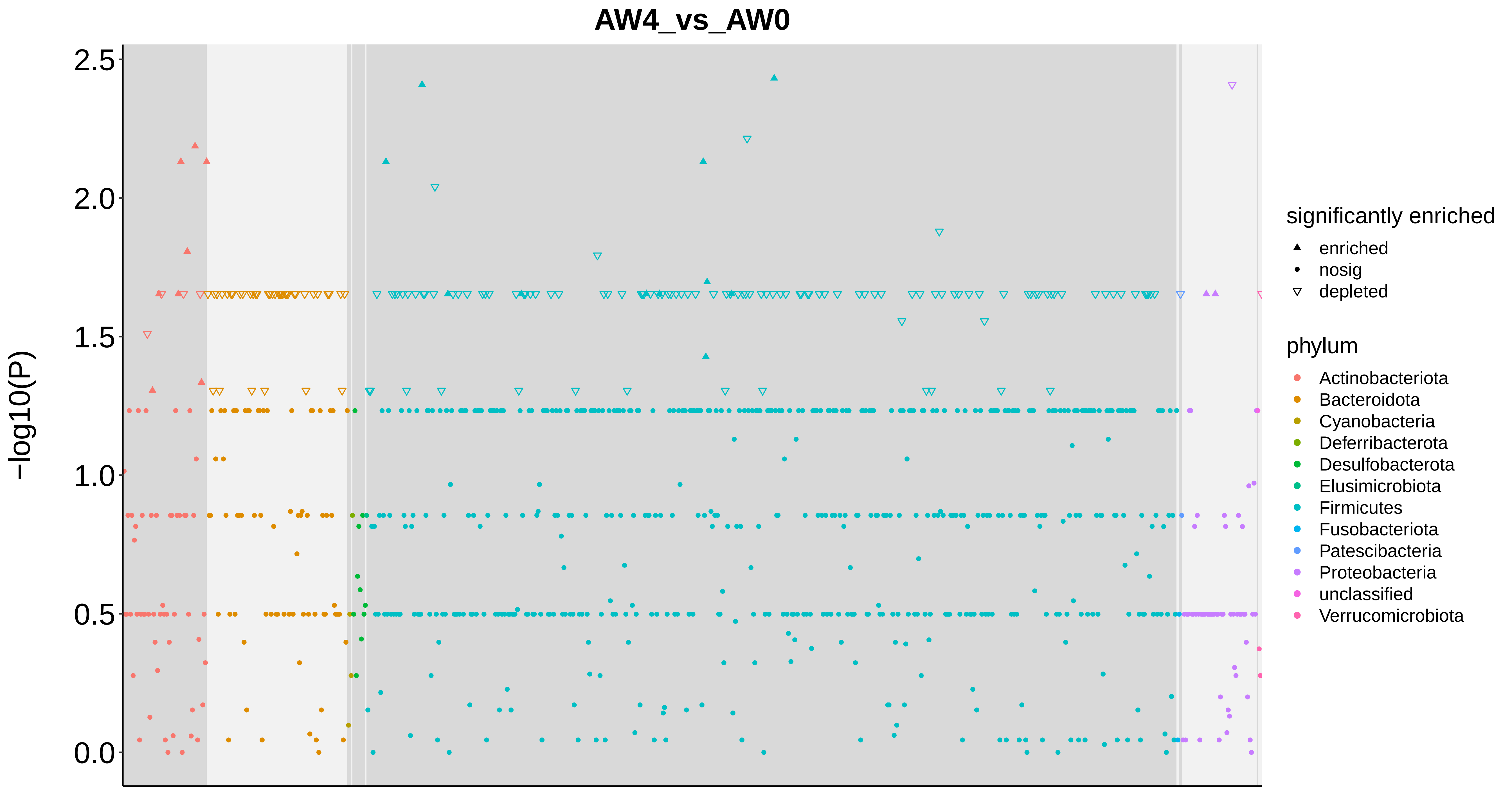

AW4_vs_AW0
-log10(P)
0.0
0.5
1.0
1.5
2.0
2.5
significantly enriched
enriched
nosig
depleted
phylum
Actinobacteriota
Bacteroidota
Cyanobacteria
Deferribacterota
Desulfobacterota
Elusimicrobiota
Firmicutes
Fusobacteriota
Patescibacteria
Proteobacteria
unclassified
Verrucomicrobiota
